# Topology Optimization for Maximizing the Fracture Resistance of Periodic Quasi-Brittle Composites Structures

**DOI:** 10.3390/ma13153279

**Published:** 2020-07-23

**Authors:** Daicong Da, Julien Yvonnet

**Affiliations:** MSME, Université Gustave Eiffel, CNRS UMR 8208, F-77474 Marne-la-Vallée, France; daicong.da@u-pem.fr

**Keywords:** topological optimization, phase field model, fracture, interfacial cracking, fracture resistance, multiple objectives, periodic composites

## Abstract

Topology optimization for maximizing the fracture resistance of particle-matrix composites is investigated. The methodology developed in our previous works, combining evolutionary topology optimization and phase field method to fracture embedding interfacial damage, is applied and extended to periodic composites and multiple objectives. On one hand, we constrain the periodicity of unit cells geometry and conduct their topology optimization for one given load prescribed over the whole structure. On the other hand, we consider a single unit cell whose topology is optimized with respect to the fracture energy criterion when subjected to multiple loads. Size effects are investigated. We show that significant enhancement of the fracture resistance can be achieved for the studied composite structures by the present method. In addition, a first attempt to fracture resistance enhancement of a unit cell associated with a material is investigated for multiple loads, exhibiting a complex optimized microstructure.

## 1. Introduction

Over the past few decades, topology optimization (TO) has undergone a tremendous development not only regarding academic investigations but also in various industrial applications since the seminal paper in [1]. The key merit of topology optimization over conventional size and shape optimization is that the former can provide more design freedom, consequently leading to the creation of novel and highly efficient designs. With the increasing development of contemporary manufacturing processes such as the popular additive manufacturing/3D printing techniques [2], it is nowadays possible to directly fabricate topologically optimized designs from digital files with arbitrary geometrical shapes.

**Figure 1:**
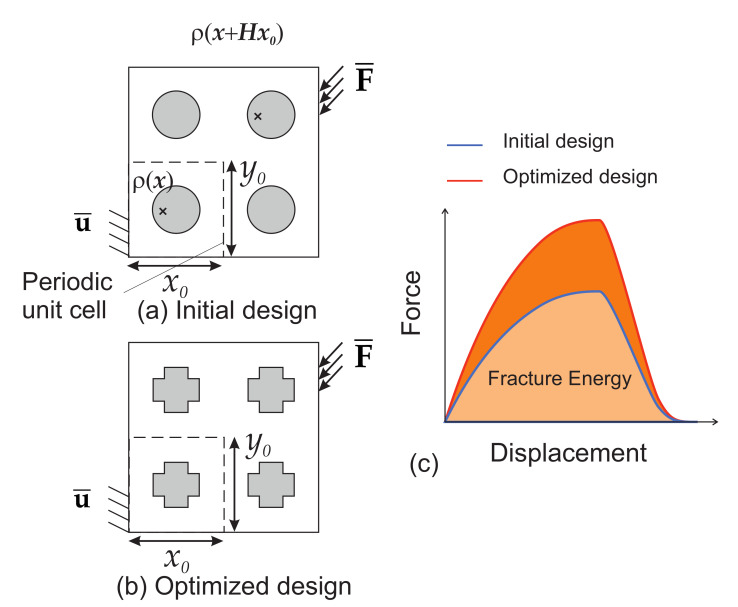
Schematic view of the methodology: the topology of the periodic unit cell is optimized such as the fracture energy of the whole structure is maximized through the whole fracturing process, from crack initiation until complete failure of the structure: (**a**) initial design; (**b**) optimized design; (**c**) load–displacement response of the structure for initial and optimized designs.

Topology optimization in the context of minimizing structural compliance and maximizing the elastic mechanical properties of composite materials has been extensively studied [3,4,5,6,7,8,9,10,11]. In most cited works, only linear properties have been investigated. One new and challenging topic which has emerged recently is TO for maximizing fracture resistance of structures and materials. Fracture resistance to mechanical load is amongst the most important criteria to design a structure in engineering and the use of TO to help designing structures and materials with respect to this criterion is of critical importance. By resistance to fracture, we mean here maximizing the total mechanical work during the whole fracturing process, from initiation up to propagation of internal micro cracks and finally failure of the structure (see Figure 1). This is in contrast with, e.g., maximizing limit stress state in an elastic context, as it requires simulating the full nonlinear fracturing process within the optimization scheme.

Early works attempting to maximize fracture resistance using TO can be traced back to [12] in the context of Level-set TO and linear fracture mechanics, and have been recently developed in a similar framework in [13]. Improvements of this idea by taking into account an incremental damage response during the load have been proposed, incorporating damage mechanics [14,15], nonlocal damage theory [16], or interfacial damage [17].

Another important progress was to include brittle fracture propagation within TO. First attempts have made use of fracture mechanics like XFEM [18], but such approaches require the existence of an initial crack and are limited to moderately simple crack morphologies. The phase field method [19,20,21,22,23,24,25,26,27,28], has many advantages to describe brittle fractures while maintaining the simplicity and versatility of simple finite elements, and allows for initiating cracks, handling complex and arbitrary, 3D crack networks and can be extended to more complex behaviors like plasticity [29], interfacial damage [28,30], or coupled multiphysics [31], among many others. Another very important point is that, within phase field, crack paths are not mesh-dependent, and a regular mesh can be employed, which might be advantageous in TO procedures.

Phase field and genetic algorithms have been used in [32] to find the optimal location of particles to maximize fracture mechanics indicators such as the peak force, maximum deformation at failure point, and maximum fracture energy during an incremental procedure. The first works to our best knowledge combining phase field and TO were introduced in [33,34,35], where the Bi-directional Evolutionary Structural Optimization (BESO) TO [36] was employed to optimize the fracture resistance of two-phase structures with respect to inclusion shapes, including cracks in both bulk and interfaces. In [37,38], Solid Isotropic Material with Penalization (SIMP) TO and phase field were combined to optimize the fracture energy in one-phase material structures, and a Level-Set TO-phase field approach was proposed in [39] to optimize the fracture resistance of composites. A SIMP-phase field procedure for two-phase materials with comparison with BESO has been developed in [40]. An experimental study involving the fracture of 3D-printed topology-optimized beams can be found in [41]. A comparative review of the different TO approaches can be found in [10].

This paper presents developments of the framework proposed in [34], combining BESO topology optimization and the phase field method to fracture embedding interfacial damage. The contributions include extensions to periodic composites, where the topology optimization is constrained to be periodic over the different unit cells composing the structure. Another extension to multiple loads is proposed, applied to the fracture resistance of one unit cell subjected to possible multiple loads. Finally, we investigate strength size effects in the optimized periodic composite structures.

The paper is organized as follows. We first review the phase field method to fracture in Section 2. The topology optimization framework and the introduced extensions to maximize the fracture resistance, combining the incremental simulations and BESO method, is presented in Section 3. Numerical examples are provided in Section 4, to show the potential of the method and to investigate strength size effects in such optimized structures.

## 2. Phase Field Modeling of Bulk Crack and Interfaces

In this section, we briefly review the phase field model for fractures interacting with interfacial damage as presented in [28]. This model will basically serve as an engine for the topology optimization procedure. Linearization and implementation aspects are briefly reviewed in Appendix A and Appendix B. More details can be found in our previous works [28,34].

**Figure 2:**
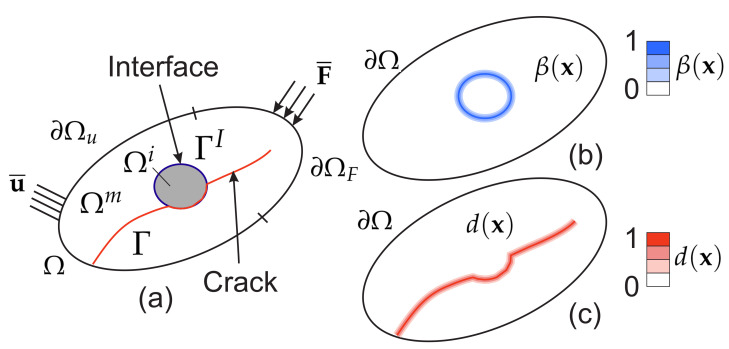
(**a**) cracked structure: boundary conditions and sharp description of cracks and interfaces; (**b**) smoothed description of the interfaces through the field β(x); (**c**) smoothed description of cracks through the field d(x).

Let $\Omega \in R^{d}$ be an open domain describing a solid with external boundary ∂Ω (see Figure 2a). The solid contains internal material interfaces between different phases, collectively denoted by $\Gamma^{I}$, as illustrated in Figure 2a. The heterogeneous material is composed of two phases, the matrix and inclusions, associated respectively to the domains $\Omega^{m}$ and $\Omega^{i}$, and such that $\Omega =\Omega^{m}\cup \Omega^{i}$. During the loading, cracks may propagate within the solid and can pass through the material interfaces. The crack surfaces are collectively denoted by Γ. In this work, we adopt smeared representations of both cracks and material interfaces: the interfaces between different material phases are described by a fixed scalar phase field β(x) (see Figure 2b) while cracks are approximated by an evolving phase field d(x,t) (see Figure 2c). The regularized parameters describing the actual widths of the smeared cracks and material interfaces are respectively denoted by $\ell_{d}$ and $\ell_{\beta }$. In the following, the same regularization length $\ell =\ell_{\beta }=\ell_{d}$ is adopted for cracks and material interfaces for the sake of simplicity. The macroscopic energy of a structure containing sharp cracks and interfaces is defined by (see e.g., [28]):

(1) $$E=\int_{\Omega }\Psi^{e}\left(\varepsilon (u)\right)d\Omega +\int_{\Gamma }g_{c}d\Gamma +\int_{\Gamma^{I}}\Psi^{I}\left(\left[\;\left[u\right]\;\right]\right)d\Gamma ,$$

where $\Psi^{e}$ is the elastic bulk strain energy density, ε is the second-order strain tensor, u is the displacement field, $\Psi^{I}$ is an interface strain density function, $g_{c}$ is the toughness, and $\left[\;\left[u\right]\;\right]$ is the displacement jump across the interface $\Gamma^{I}$.

In [28], we have proposed a smoothed representation of both discontinuities associated with bulk and interface cracks. In this framework, the above energy functional is substituted by the following expression:

(2) $$E=\int_{\Omega }\Psi^{e}\left(\varepsilon^{e}\left(u,d,\beta \right)\right)d\Omega +\int_{\Omega }\left[1-\beta \left(x\right)\right]g_{c}\gamma_{d}\left(d\right)d\Omega +\int_{\Omega }\Psi^{I}\left(w\right)\gamma_{\beta }\left(\beta \right)d\Omega ,$$

where $\gamma^{d}$ is a crack density function associated with the smoothed representation of bulk cracks defined by

(3) $$\begin{array}{c} \gamma_{d}\left(d(x)\right)=\frac{1}{2\ell_{d}}d{\left(x\right)}^{2}+\frac{\ell_{d}}{2}\nabla d\left(x\right)\cdot \nabla d\left(x\right). \end{array}$$

In (2), $\Psi^{e}$ refers to the strain energy density function depending on the part of the strain $\varepsilon^{e}$ defined by:

(4) $$\varepsilon^{e}=\nabla^{s}u-n{⊗}^{S}w\gamma_{\beta }$$

where ${\left(\nabla^{s}u\right)}_{ij}=\left(u_{i,j}+u_{j,i}\right)/2$ and ${\left(n{⊗}^{S}w\right)}_{ij}=\left(n_{i}w_{j}+w_{i}n_{j}\right)/2$. The strain density function is defined such that the damage is associated with tensile strain only and is given in the form:

(5) $$\Psi^{e}=\left(1-d\right)\Psi^{e+}+\Psi^{e-},$$

where $\Psi^{e+}$ and $\Psi^{e-}$ denote the parts of the elastic strain density function associated with tensile and compressive parts, respectively, and are expressed for an isotropic material as:

(6) $$\Psi_{e}^{\pm }\left(\varepsilon \right)=\lambda {\langle \mathrm{tr}\left[\varepsilon^{e}\right]\rangle }_{\pm }^{2}/2+\mu \mathrm{tr}{\left[\varepsilon^{e\pm }\right]}^{2}.$$

Above, $\varepsilon^{e\pm }$ are the tensile and compressive parts of the strain tensor, respectively, defined in the following (see Equation (13)) and λ and μ are the Lamé’s elastic parameters. Note that we have omitted the dependance of the local parameters to the local phase, i.e., the local elasticity parameters are defined as $\lambda^{i}$, $\mu^{i}$ and $\lambda^{m}$, $\mu^{m}$, where the indices *i* and *m* refer to the inclusion and matrix phase materials, respectively. Similarly, local toughness are denoted by $g_{c}^{i}$ and $g_{c}^{m}$, respectively.

The field β(x) is a smooth indicator for interfaces which satisfies:

(7) $$\left\{\begin{array}{c} \beta \left(x\right)-\ell_{\beta }^{2}\left(x\right)▵\beta \left(x\right)=0\;\;\mathrm{in}\;\Omega ,\\ \beta \left(x\right)=1\;\;\mathrm{on}\;\Gamma^{I},\\ \nabla \beta (x)\cdot n=0\;\;\mathrm{on}\;\partial \Omega , \end{array}\right.$$

where $\ell_{\beta }$ is the regularization parameter describing the width of the regularized interfaces, with

(8) $$\begin{array}{c} \gamma_{\beta }\left(\beta \right)=\frac{1}{2\ell_{\beta }}\beta {\left(x\right)}^{2}+\frac{\ell_{\beta }}{2}\nabla \beta \left(x\right)\cdot \nabla \beta \left(x\right). \end{array}$$

Problem (7) can be solved by classical finite elements (see [28]). Above, w(x) is a smooth representation of the displacement jump function across the interface, defined by:

(9) $$w\left(x\right)≃\left[\;\left[u(x)\right]\;\right]≃u\left(x+\frac{h}{2}n^{I}\right)-u\left(x-\frac{h}{2}n^{I}\right)=h\nabla u\left(x\right)\frac{\nabla \phi (x)}{∥\nabla \phi (x)∥}.$$

Using variational principle for minimizing *E* with respect to displacements (assuming the crack phase field *d* and interface β are fixed), i.e.,

(10) $$u\left(x\right)=Arg\left\{\underset{u\in S_{u}}{\inf}\left(E(u,d,\beta )-W^{ext}\right)\right\},$$

where $S_{u}=\left\{u|u\left(x\right)=\bar{u}\;\mathrm{on}\;\partial \Omega_{u},\;\;u\in H^{1}\left(\Omega \right)\right\}$ and $W^{ext}=\int_{\Omega }f\cdot ud\Omega +\int_{\partial \Omega_{F}}\bar{F}\cdot ud\Gamma$ with f and $\bar{F}$ being body forces and prescribed traction over the boundary $\partial \Omega_{F}$, we obtain the weak form for $u\left(x\right)\in S_{u}$:

(11) $$R_{1}=\int_{\Omega }\sigma^{e}(u,d):\varepsilon^{e}\left(\delta u\right)+t(w(u))\cdot \delta w(\delta u)\gamma_{\beta }\left(\beta \right)d\Omega -\int_{\partial \Omega_{F}}\bar{F}\cdot \delta ud\Gamma -\int_{\Omega }f\cdot \delta ud\Omega =0,$$

with $\delta u\in S_{u}^{0}$, $S_{u}^{0}=\left\{v|v\left(x\right)=0\;\mathrm{on}\;\partial \Omega_{u},\;\;v\in H^{1}\left(\Omega \right)\right\}$ and where the Cauchy stress $\sigma^{e}$ is expressed as

(12) $$\sigma^{e}=\left({\left(1-d\right)}^{2}+k\right)\left\{\lambda {\langle Tr\varepsilon^{e}\rangle }_{+}1+2\mu \varepsilon^{e+}\right\}+\lambda {\langle Tr\varepsilon^{e}\rangle }_{-}1+2\mu \varepsilon^{e-},$$

with k<<1 a small scalar parameter, $\varepsilon^{e}\left(\delta u\right)=\nabla^{s}\delta u-n{⊗}^{S}w\gamma_{\beta }$ and δw=h∇(δu)∇ϕ/∥∇ϕ∥. Above, $\varepsilon^{e\pm }$ are defined according to:

(13) $$\varepsilon^{e\pm }=\sum_{i=1}^{D}{\langle \varepsilon^{i}\rangle }_{\pm }n^{i}⊗n^{i},$$

where $\varepsilon^{i}$ and $n^{i}$ are the eigenvalues and eigenvectors of $\varepsilon^{e}$, i.e., satisfying $\varepsilon^{e}n^{i}=\varepsilon^{i}n^{i}$. In (12) and (13), ${\langle x\rangle }_{\pm }=\left(x\pm \left|x\right|\right)/2$. The cohesive traction law at the interfaces is given in 2D by:

(14) $$t\left(w\right)={\left[t^{n},\;t^{t}\right]}^{T}$$

where $t^{n}$ and $t^{t}$ denote normal and tangential parts of the traction vector t across the interface $\Gamma^{I}$ oriented by its normal $n^{I}$. We chose the simple model [42]:

(15) $$t^{n}=g_{c}^{I}\frac{w^{n}}{{\left(\delta^{n}\right)}^{2}}\exp\left(-\frac{w^{n}}{\delta^{n}}\right),$$

where $\delta^{n}$ is related to the interface fracture toughness $g_{c}^{I}$ and the interface fracture strength $t_{u}$ by:

(16) $$\delta^{n}=\frac{g_{c}^{I}}{t_{u}\exp\left(1\right)},$$

$w^{n}=w\cdot n^{I}$, $t^{t}=0$ for the sake of simplicity, and $g_{c}^{I}$ is the toughness associated with the interface. Note that the problem (11) is nonlinear. A Newton iterative scheme is then required to solve it, as described in Appendix A.

The phase field problem is obtained by minimization of energy under the constraint that $\dot{d}\geq 0$. Here, we follow the approach of Miehe et al. [23] and extended in [28] where the weak form associated with this process is given by: find $d\left(x\right)\in S_{d}$, $S_{d}=\left\{d|d\left(x\right)=1\;\mathrm{on}\;\Gamma ,\;\;d\in H^{1}\left(\Omega \right)\right\}$:

(17) $$R_{2}=\int_{\Omega }\left\{\left(2H+\left[1-\beta \right]\frac{g_{c}}{\ell_{d}}\right)d\delta d+\left[1-\beta \right]g_{c}\ell_{d}\nabla d\cdot \nabla \left(\delta d\right)\right\}d\Omega -\int_{\Omega }2H\delta dd\Omega =0,$$

and $\delta d\left(x\right)\in S_{d}^{0}$, $S_{d}^{0}=\left\{\delta d|\delta d\left(x\right)=0\;\mathrm{on}\;\partial \Omega ,\;\;d\in H^{1}\left(\Omega \right)\right\}$ and where the history functional is introduced to handle the Irreversibility condition:

(18) $$H(x,t)=\underset{\tau \in \left[0,t\right]}{max}\left\{\Psi_{e}^{+}\left(x,\tau \right)\right\}.$$

In the present work, we used a staggered procedure to solve alternatively problems (11) and (17). Numerical details and finite element discretization of the mechanical problem and phase field problem are provided in Appendix A and Appendix B, respectively. The overall algorithm is summarized as follows.

1. Set the initial displacement field $u_{0}\left(x\right)$, the phase field $d_{0}\left(x\right)$, and the strain-history function $H_{0}$.
2. **For** all loading increments: (at each time $t_{n+1}$), given $d_{n},u_{n},$ and $H_{n}\left(x\right)$.
  (a) Compute the history function $H(t_{n+1})$ according to (18).
  (b) Compute the crack phase field $d_{n+1}\left(x\right)$ by solving phase field problem (17).
  (c) Compute $u_{n+1}\left(x\right)$ :
    - Initialize $u_{k}=u_{n}$
      **While**$||\Delta u_{k+1}\left|\right|>\epsilon ,\epsilon \ll 1$:
    - Compute $\Delta u_{k+1}^{e}$ by solving linearized mechanical problem (A14).
    - Update $u_{k+1}=u_{k}+\Delta u_{k+1}^{e}$.
    - ${\left(.\right)}_{n+1}\to {\left(.\right)}_{n}$ and go to (a).
**End**
**End**

## 3. Topology Optimization Problem to Fracture Resistance Maximization

In this section, the topology optimization problem to fracture resistance maximization, first defined in [33,34], is extended here to periodic composite structures and multiple objectives.

### 3.1. Problem Statement

#### 3.1.1. Periodic Structures

The topology optimization problem is conducted with respect to a density variable ρ(x) which is associated with the inclusion phase. In other words, $\rho \left(x\right)=1\;\forall x\in \Omega^{i}$ and $\rho \left(x\right)=0\;\forall x\in \Omega^{m}$ (see Figure 2a). We consider periodic structures composed of a repetition of $N_{x}\times N_{y}$ unit cells along x− and y− directions. The dimensions of the unit cell are $x_{0}\times y_{0}$. The optimization problem is then defined as follows:

(19) $$\begin{array}{cc} \mathrm{Maximize}:\; & J(\rho ,u,d)\\ \rho (x) & \\ u\left(t\right)\in S_{u} & \\ d\left(t\right)\in S_{d} & \end{array}$$

(20) $$\begin{array}{cc} \mathrm{subject}:\; & R_{1}\left(\rho ,u(t),d(t)\right)=0,\;t\in \left[0,t^{max}\right] \end{array}$$

(21) $$\begin{array}{cc} \; & R_{2}\left(\rho ,u(t),d(t)\right)=0,\;t\in \left[0,t^{max}\right] \end{array}$$

(22) $$\begin{array}{cc} \; & \bar{f}=\frac{Vol(\Omega^{i})}{Vol(\Omega )}=\frac{\int_{\Omega }\rho \left(x\right)d\Omega }{Vol(\Omega )}, \end{array}$$

(23) $$\begin{array}{cc} \; & \rho \left(x\right)=\rho \left(x+Hx_{0}\right),\; \end{array}$$

with

(24) $$J=\int_{0}^{t^{max}}f^{ext}\left(t\right)\cdot \bar{U}\left(t\right)dt,$$

where $R_{1}$ is given by (11) and $R_{2}$ is given by (17), $t^{max}$ denotes the maximum simulation pseudo-time associated with the final prescribed displacement at the failure step, $f^{ext}$ is the external force response at the load point, and $\bar{f}$ is the prescribed volume fraction of the inclusion phase and $\bar{U}$ is the prescribed displacement load vector. Above, $S_{u}$ and $S_{d}$ are the appropriate vector spaces associated with the unknown fields u and *d* defined in Section 2. The objective function is the total mechanical work during the fracturing process. Above, H=[i0;0j], $i=1,2,\ldots ,N_{x}$, j=1,2,…,Ny is a matrix which defines the periodicity with respect to the unit cell and $x_{0}$ is a point coordinate in the periodic unit cell Ω (see Figure 1). The pseudo-density ρ can be interpreted as an indicator such that the value of one corresponds to the inclusion phase, whereas zero corresponds to the matrix phase. Note that, in the present framework, quasi-static simulations are performed, and the time is here only related to the load evolution. In addition, the elasticity parameters and toughness of the phases are defined with respect to the density as:

(25) $$\lambda \left(\rho \right)=\rho \lambda^{i}+\left(1-\rho \right)\lambda^{m},\;\;\mu \left(\rho \right)=\rho \mu^{i}+\left(1-\rho \right)\mu^{m},$$

(26) $$g_{c}\left(\rho \right)=\rho g_{c}^{i}+\left(1-\rho \right)g_{c}^{m}.$$

Note that evaluation of (24) requires solving the full fracture problem, from initiation to complete failure of the structure.

#### 3.1.2. Multiple Objectives

In the case when the topology is optimized with respect to k=1,2,…,N loads, the optimization problem is modified according to:

(27) $$\begin{array}{cc} \mathrm{Maximize}:\; & \tilde{J}\left(\rho ,{u^{k}}_{i},{d^{k}}_{i}\right)\\ \rho (x) & \\ u_{k}\left(t\right)\in S_{u} & \\ d_{k}\left(t\right)\in S_{d} & \\ k=1,2,\ldots ,N & \end{array}$$

(28) $$\begin{array}{cc} \mathrm{subject}:\; & R_{1}^{k}\left(\rho ,u^{k}\left(t\right),d^{k}\left(t\right)\right)=0,\;t\in \left[0,t^{max}\right],\;k=1,2,\ldots ,N \end{array}$$

(29) $$\begin{array}{cc} \; & R_{2}^{k}\left(\rho ,u^{k}\left(t\right),d^{k}\left(t\right)\right)=0,\;t\in \left[0,t^{max}\right],\;k=1,2,\ldots ,N \end{array}$$

(30) $$\begin{array}{cc} \; & \bar{f}=\frac{Vol(\Omega^{i})}{Vol(\Omega )}=\frac{\int_{\Omega }\rho \left(x\right)d\Omega }{Vol(\Omega )}, \end{array}$$

(31) $$\begin{array}{cc} \; & \rho \left(x\right)=\rho \left(x+Hx_{0}\right), \end{array}$$

(32) $$\tilde{J}=\sum_{k=1}^{N}J_{k},$$

and

(33) $$J_{k}=\int_{0}^{t_{k}^{max}}f^{ext}\left(t\right)\cdot \bar{U}\left(t\right)dt$$

with $t_{k}^{max}$ the maximum simulation time for the k−th load.

### 3.2. Discrete Topology Optimization Problem

In what follows, we only define the solution procedure for the periodic composite problem, as the idea is very similar for the multiple loading case. Problems (19)–(24) are solved using Finite Elements based on the same mesh than the one used to solve the displacement and fracture problems defined in Section 2. A regular mesh of bi-linear 4-node elements is employed.

We define the vector of discrete density values $\left\{\rho \right\}$= $\left\{\rho_{1},\rho_{2},\ldots ,\rho_{N_{e}}\right\}$ with $N_{e}$ the number of Finite Elements. The discrete form of (22) and (23) is then defined as:

(34) $$\begin{array}{cc} \mathrm{Maximize}:\; & J^{h}\left(\left\{\rho \right\},u,d\right)\\ \left\{\rho \right\} & \\ u\left(t\right)\in S_{u} & \\ d\left(t\right)\in S_{d} & \end{array}$$

(35) $$\begin{array}{cc} \mathrm{subject}:\; & R_{1}\left(\left\{\rho \right\},u\left(t^{n}\right),d\left(t^{n}\right)\right)=0,\;n=1,2,\ldots ,n_{load} \end{array}$$

(36) $$\begin{array}{cc} \; & R_{2}\left(\left\{\rho \right\},u\left(t^{n}\right),d\left(t^{n}\right)\right)=0,\;n=1,2,\ldots ,n_{load} \end{array}$$

(37) $$\begin{array}{cc} \; & \sum_{e=1}^{N_{e}}\rho_{e}v_{e}/\left(\sum_{e=1}v_{e}\right)=\bar{f} \end{array}$$

(38) $$\begin{array}{cc} \; & \rho_{e}\left(x\right)=\rho_{e}\left(x+Hx_{0}\right) \end{array}$$

where $n_{load}$ is the number of load steps and $J^{h}$ in (34) is approximated by:

(39) $$J\approx \frac{1}{2}\sum_{n=1}^{n_{\mathrm{load}}}{\left(f_{\mathrm{ext}}^{\left(n\right)}+f_{\mathrm{ext}}^{\left(n-1\right)}\right)}^{T}\Delta u^{\left(n\right)},$$

where $\Delta u^{\left(n\right)}$ denotes the prescribed load increment at load *n* and $R_{1}$ is approximated by

(40) $$R_{1}=f_{\mathrm{ext}}-f_{\mathrm{int}}.$$

### 3.3. Sensitivity Analysis and Extended BESO Procedure

In order to perform the topology optimization, the sensitivity of the objective function *J* with respect to topology design variables $\left\{\rho \right\}$ must be computed. The adjoint method is employed to derive the sensitivity analysis. Firstly, two adjoint vectors $\mu^{\left(n\right)}$, $\lambda^{\left(n\right)}$ of the same dimension as the vector of unknowns u are introduced to enforce zero residual $R_{1}$ at load steps n−1 and *n* for each term of the total mechanical work (39). To be solved by the adjoint method, the modified expression for the objective function is introduced as follows:

(41) $$\hat{J}=\frac{1}{2}\sum_{n=1}^{n_{\mathrm{load}}}\left\{{\left(f_{\mathrm{ext}}^{\left(n\right)}+f_{\mathrm{ext}}^{\left(n-1\right)}\right)}^{T}\Delta u^{\left(n\right)}+{\left(\lambda^{\left(n\right)}\right)}^{T}{R_{1}}^{\left(n\right)}+{\left(\mu^{\left(n\right)}\right)}^{T}{R_{1}}^{\left(n-1\right)}\right\}.$$

Due to the asserted static equilibrium, the residuals ${R_{1}}^{\left(n\right)}$ and ${R_{1}}^{\left(n-1\right)}$ must vanish. The objective value is thus invariant with respect to the values of the adjoint vectors $\lambda^{\left(n\right)}$ and $\mu^{\left(n\right)}$ ($n=1,\ldots ,n_{\mathrm{load}}$), i.e.,

(42) $$\hat{J}\left(\rho ;{\left\{\lambda^{\left(n\right)},\mu^{\left(n\right)}\right\}}_{n=1,\ldots ,n_{\mathrm{load}}}\right)=J\left(\rho \right).$$

This equivalence also holds for the sensitivity with respect to changes of density $\rho_{e}$ on element *e*

(43) $$\frac{\partial \hat{J}}{\partial \rho_{e}}=\frac{\partial J}{\partial \rho_{e}}.$$

In the following, the derivative $\partial \hat{J}/\partial \rho_{e}$ is computed with properly determined values of $\lambda^{\left(n\right)}$ and $\mu^{\left(n\right)}$ leading to simplifications in the derivation. To formally describe these derivations, we introduce a partitioning of all degrees of freedom (DOF) into essential (index E; associated with Dirichlet boundary conditions) and free (index F; remaining DOF) entries. For a vector a and a matrix M, we have

(44) $$a∼\left[\begin{array}{c} a_{E}\\ a_{F} \end{array}\right]\;\;\;\;\mathrm{and}\;\;\;\;M∼\left[\begin{array}{cc} M_{\mathrm{EE}} & M_{\mathrm{EF}}\\ M_{\mathrm{FE}} & M_{\mathrm{FF}} \end{array}\right].$$

It can be shown (see e.g., [35]) that

(45) $$\begin{array}{cc} \frac{\partial \hat{J}}{\partial \rho_{e}}=\frac{1}{2}\sum_{n=1}^{n_{\mathrm{load}}}\{ & -{\left(\lambda^{\left(n\right)}\right)}^{T}\int_{\Omega_{i}}B^{T}{\left(\sigma^{e}\right)}^{\left(n\right)}d\Omega^{e}-{\left(\mu^{\left(n\right)}\right)}^{T}\int_{\Omega_{i}}B^{T}{\left(\sigma^{e}\right)}^{\left(n-1\right)}d\Omega_{i}\\ & -{\left(K_{\tan,\mathrm{FE}}^{\left(n\right)}\lambda_{E}^{\left(n\right)}+K_{\tan,\mathrm{FF}}^{\left(n\right)}\lambda_{F}^{\left(n\right)}\right)}^{T}\frac{\partial \Delta u_{F}^{\left(n\right)}}{\partial \rho_{i}}\\ & -{\left(K_{\tan,\mathrm{FE}}^{\left(n-1\right)}\mu_{E}^{\left(n\right)}+K_{\tan,\mathrm{FF}}^{\left(n-1\right)}\mu_{F}^{\left(n\right)}\right)}^{T}\frac{\partial \Delta u_{F}^{\left(n-1\right)}}{\partial \rho_{i}}\}, \end{array}$$

where

(46) $$K_{\tan}^{\left(m\right)}=-\frac{\partial {R_{1}}^{\left(m\right)}}{\partial u^{\left(m\right)}}$$

is the tangent stiffness matrix of the nonlinear mechanical system at the balance equation of the m−th load increment.

To avoid the evaluation of the unknown derivatives of $u_{F}^{\left(n\right)}$ and $u_{F}^{\left(n-1\right)}$, i.e., eliminating the last two lines of (45), the values of $\lambda_{F}^{\left(n\right)}$ and $\mu_{F}^{\left(n\right)}$ are sought as following by solving the adjoint systems with the prescribed values at the essential nodes:

(47) $$\lambda_{E}^{\left(n\right)}=-\Delta u_{E}^{\left(n\right)},\;\;\mu_{E}^{\left(n\right)}=-\Delta u_{E}^{\left(n\right)}$$

and

(48) $$\lambda_{F}^{\left(n\right)}={\left(K_{\tan,\mathrm{FF}}^{\left(n\right)}\right)}^{-1}K_{\tan,\mathrm{FE}}^{\left(n\right)}\Delta u_{E}^{\left(n\right)},$$

and

(49) $$\mu_{F}^{\left(n\right)}={\left(K_{\tan,\mathrm{FF}}^{\left(n-1\right)}\right)}^{-1}K_{\tan,\mathrm{FE}}^{\left(n-1\right)}\Delta u_{E}^{\left(n\right)}.$$

The two relations (48) and (49) together with (47) fully determine the values of the adjoint vectors $\lambda^{\left(n\right)}$ and $\mu^{\left(n\right)}$. Finally, the objective function $\partial \hat{J}/\partial \rho_{e}$ can be computed via

(50) $$\frac{\partial \hat{J}}{\partial \rho_{e}}=-\frac{1}{2}\sum_{n=1}^{n_{\mathrm{load}}}\left\{{\left(\lambda^{\left(n\right)}\right)}^{T}\int_{\Omega_{i}}B^{T}{\left(\sigma^{e}\right)}^{\left(n\right)}d\Omega^{e}+{\left(\mu^{\left(n\right)}\right)}^{T}\int_{\Omega^{e}}B^{T}{\left(\sigma^{e}\right)}^{\left(n-1\right)}d\Omega_{i}\right\}.$$

The computation of the sensitivity consists in solving two linear systems of Equations (48) and (49).

The extended BESO method recently developed in [43,44] using an additional damping treatment on sensitivity numbers is adopted in this work. It has been shown that this treatment can improve the robustness and efficiency of the method, especially in dealing with nonlinear designs in the presence of dissipative effects. Note that, in the present work, due to the staggered approach used to solve the problems (11) and (17), we only take into account the constraint (20) through the sensitivity analysis, assuming that (17) is verified at the previous iteration. Details about BESO implementation can be found in [34].

## 4. Numerical Examples

In all following numerical examples, bi-material composites are investigated. The material parameters of each phase are taken as $E_{i}=52$ GPa, $E_{m}=10.4$ GPa, $\nu_{i}=\nu_{m}=0.3$, where *E* denotes the Young’s modulus and ν the Poisson’s ratio. The indices *i* and *m* refer to the inclusion and matrix materials, respectively. The bulk toughness $g_{c}$ is the same for the matrix and the inclusions and equal to $g_{c}^{m}=g_{c}^{i}=g_{c}=0.1$ N/mm. The interface fracture strength is chosen as $t_{u}=10$ MPa. In addition, we use $g_{c}=g_{c}^{I}=1\times 10^{-4}$ kN/mm. In all tests, regular meshes using quadrilateral bilinear elements are adopted. The same finite element discretization is employed for both displacement and crack phase fields. The regularization parameter $\ell_{d}=\ell_{\beta }$ describing the width of bulk cracks and interface cracks is chosen as twice the finite element size $\ell_{d}=\ell_{\beta }=2\ell_{e}$, with $\ell_{e}$ the characteristic finite element size. During the topology optimization process, the volume fraction of inclusions is maintained constant. We define the gain in fracture resistance as $G=100\times \left(J^{opt}-J^{init}\right)/J^{init}$, where $J^{opt}$ is the fracture resistance of the optimized structure while $J^{init}$ is the initial structure design.

### 4.1. Periodic Composite under Three-Point Bending

**Figure 3:**
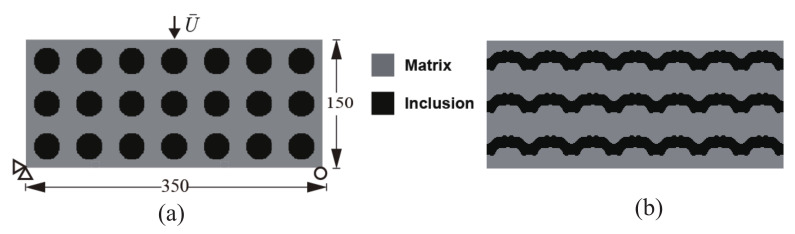
Periodic composite without initial crack subjected to three-point bending: (**a**) boundary conditions and geometry of the initial design; (**b**) final design.

We first consider the composite structure depicted in Figure 3a. The composite is made of $N_{s}=7\times 3$ periodic unit cells. The initial guess design comprises the unit cells consisting of circular inclusions in a square domain, such that the volume fraction is equal to f=30%. The dimensions of the structure are 350×150 mm${}^{2}$. The domain is uniformly discretized into 350×150 4-node bilinear elements. The boundary conditions are defined as follows: the left bottom corner node is fixed, while in the right bottom corner node, the *y*-displacement is fixed and the *x*-displacement is free. On the upper end, the load consists of a prescribed displacement $\bar{U}$ at the center point. The displacement is prescribed along the *y*-direction while the displacement along *x* is free. The computation is performed with monotonic displacement increments $\Delta \bar{U}=0.01$ mm until the corresponding resultant vertical force $F_{y}$ at the displacement application point is below a prescribed criterion value, indicating that the structure is completely broken.

As mentioned in Section 3, all unit cells are constrained to keep the same geometry during the topology optimization process. Figure 3b shows the obtained final design for this composite structure and this specific load. We observe that, while the initial design was composed of disconnected phases, the optimal design is formed with connected phases along the *x*-direction and forms a kind of wavy layered structure.

**Figure 4:**
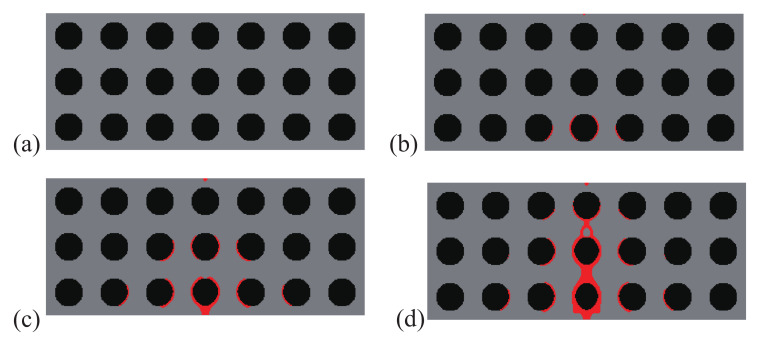
Crack propagation in the periodic composite subjected to three-point bending, initial design: (**a**) $\bar{U}=0$ mm; (**b**) $\bar{U}=0.11$ mm; (**c**) $\bar{U}=0.20$ mm; (**d**) $\bar{U}=0.28$ mm.

**Figure 5:**
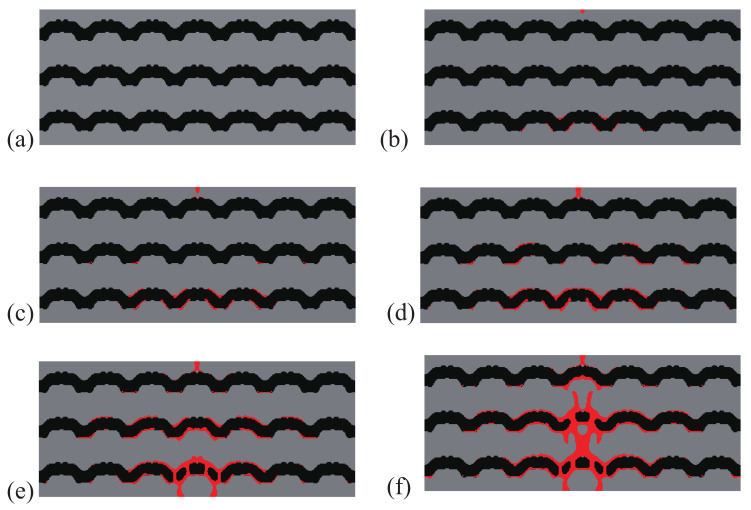
Crack propagation in the periodic composite subjected to three-point bending, optimized design: (**a**) $\bar{U}=0$ mm; (**b**) $\bar{U}=0.17$ mm; (**c**) $\bar{U}=0.23$ mm; (**d**) $\bar{U}=0.28$ mm; (**e**) $\bar{U}=0.32$ mm; (**f**) $\bar{U}=0.37$ mm.

A comparison between the crack paths obtained within the structures with initial and optimal designs are depicted in Figure 4 and Figure 5, respectively. In both cases, micro cracks nucleate from interfaces or from the surface and merge to form a macro crack, which is more complex in the case of the optimal design, with more interfacial cracks, and forming many branches, which may explain that the structure requires more energy for complete failure.

**Figure 6:**
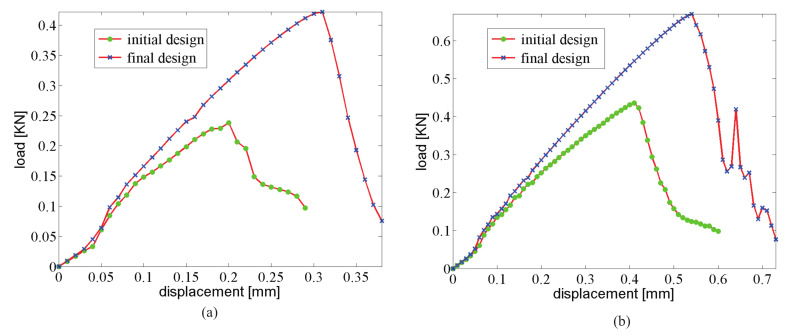
Force–displacement curve for the 3-point bending composite structure: (**a**) 350×150 mm${}^{2}$, 3×7 unit cells; (**b**) 700×300 mm${}^{2}$, 6×14 unit cells.

The displacement–force response curve of both structures with initial and optimal designs are depicted in Figure 6a. The gain in fracture resistance for the optimized structure is equal to G=124%.

### 4.2. Size Effects

In this section, size effects are investigated. We analyze the influence of the size on the strength of the structure when one fixed optimal design is used. More specifically, we use the optimal design obtained in the example of Section 4.1 and define four structures composed of 7×3, 14×6, 21×9 and 28×12 cells, corresponding to length *L* equal to 350 mm, 700 mm, 1050 mm, and 1400 mm, respectively. The associated heights *H* are 150 mm, 300 mm, 450 mm, and 600 mm. In all cases, the vertical displacement is prescribed as an increasing uniform value of $\Delta \bar{U}=0.01$ mm during the simulation, until the reaction force goes below a prescribed value indicating that the periodic composite is fully broken. One convenient way to describe the size effects in quasi-brittle structures is to plot the nominal stress $\sigma_{N}$ with respect to the height *H* in log scale. The nominal stress is defined as [45]

(51) $$\sigma_{N}=\frac{3FL}{2bH^{2}}$$

where *F* is the critical force to failure (estimated numerically in the load-displacement curve as the maximum load), *L* is the length of the beam, and *b* is an arbitrary thickness, taken here as b=1 m.

**Figure 7:**
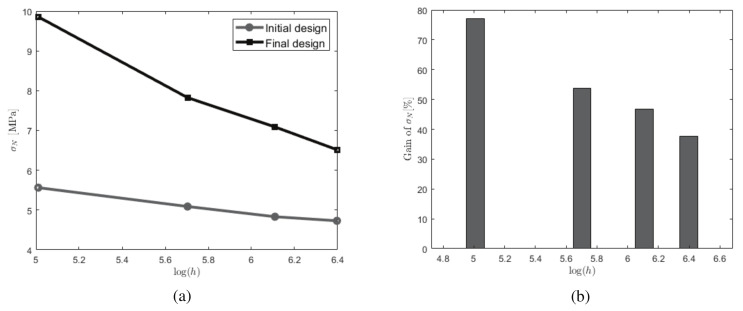
Size effects associated with the strength of the structure (**a**) nominal stress to failure with respect to the height *H* for initial and optimized topologies; (**b**) gain between initial and optimized designs with respect to the size. For each size, the same topology is kept.

Results are presented in Figure 7. The size effects are clearly shown in Figure 7a, where a decrease of nominal stress with the size is noticed. This is consistent with classical experimental results for fracture in quasi brittle beams [45]. In Figure 7b, we can see the effects of keeping the same topology and increasing the size of the structure. What is very interesting is that, when using the optimized topology obtained on the smallest structure, then, at relatively low computational costs, its use on large structure still bring a large gain (40%) in fracture resistance.

**Figure 8:**
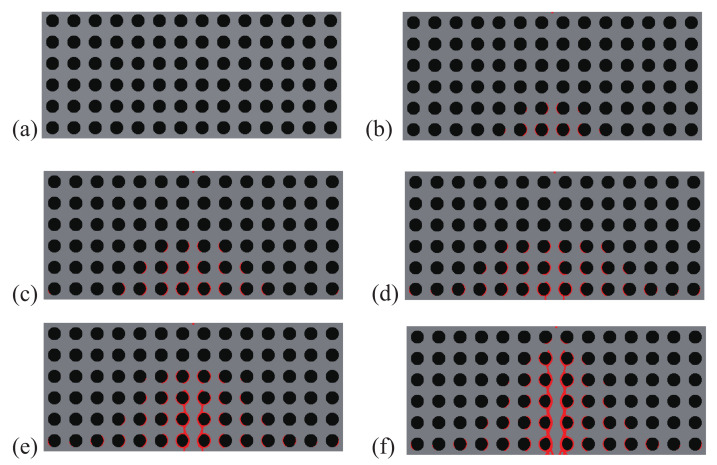
Crack propagation in the larger composite structure subjected to three-point bending, initial design: (**a**) $\bar{U}=0$ mm; (**b**) $\bar{U}=0.25$ mm; (**c**) $\bar{U}=0.35$ mm; (**d**) $\bar{U}=0.41$ mm; (**e**) $\bar{U}=0.44$ mm; (**f**) $\bar{U}=0.59$ mm.

**Figure 9:**
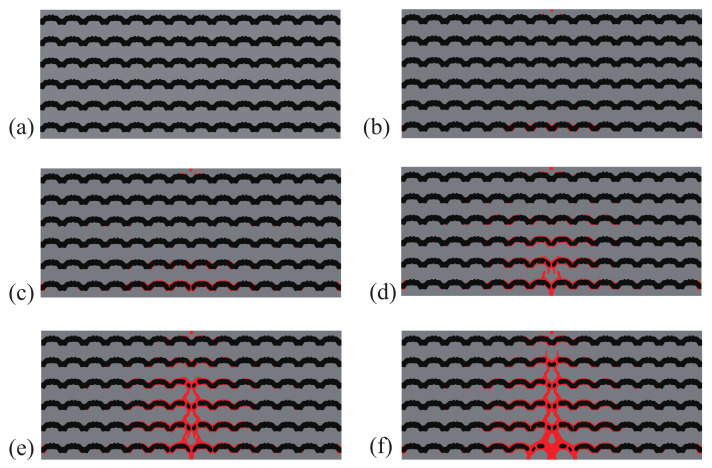
Crack propagation in the larger composite structure subjected to three-point bending, optimized design: (**a**) $\bar{U}=0$ mm; (**b**) $\bar{U}=0.39$ mm; (**c**) $\bar{U}=0.52$ mm; (**d**) $\bar{U}=0.57$ mm; (**e**) $\bar{U}=0.61$ mm; (**f**) $\bar{U}=0.72$ mm.

As an illustration, the crack propagation in both initial and optimized structures for the structure involving 6×14 cells is depicted in Figure 8 and Figure 9, respectively. We can observe the same type of patterns in both structures rather than in the smaller structure. A comparison between the reference and the optimized solution in that case is provided in Figure 6b.

### 4.3. Design of a Periodic Composite under Non-Symmetric Three-Point Bending

**Figure 10:**
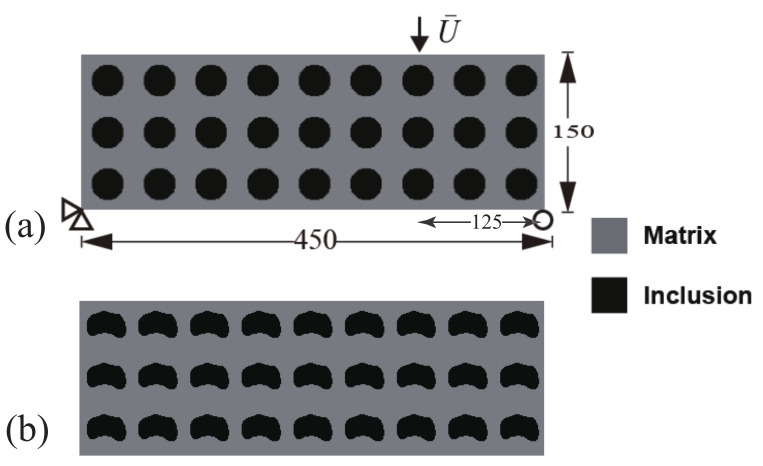
A periodic composite without initial crack subjected to non-symmetric three-point bending: (**a**) boundary conditions and geometry of the initial design; (**b**) optimized design.

We consider the same structure as in the former example, but using a non-symmetric three-point bending load. The dimensions, geometry, and boundary conditions are depicted in Figure 10a, showing the initial design, here again composed of circular inclusions as defined in the previous example. The considered composite is composed of 9×3 unit cells which are repeated periodically along each space directions. The dimension of the composite are 450×150 mm${}^{2}$, and the domain is uniformly discretized into 450×150 square shape bilinear elements. The optimized design is depicted in Figure 10b. We can see that a change in the position of the load drastically modifies the optimized design.

**Figure 11:**
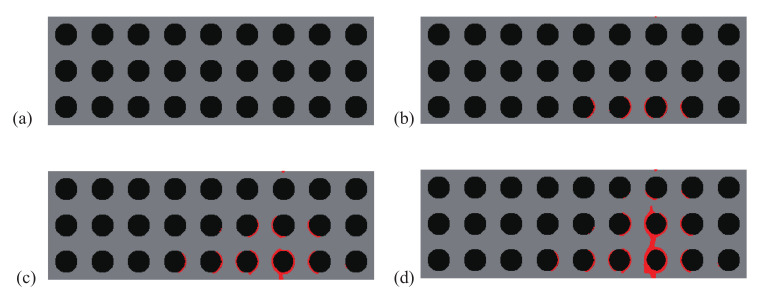
Crack propagation in the initial periodic composite subjected to non-symmetric three-point bending: (**a**) $\bar{U}=0$ mm; (**b**) $\bar{U}=0.17$ mm; (**c**) $\bar{U}=0.24$ mm; (**d**) $\bar{U}=0.30$ mm.

**Figure 12:**
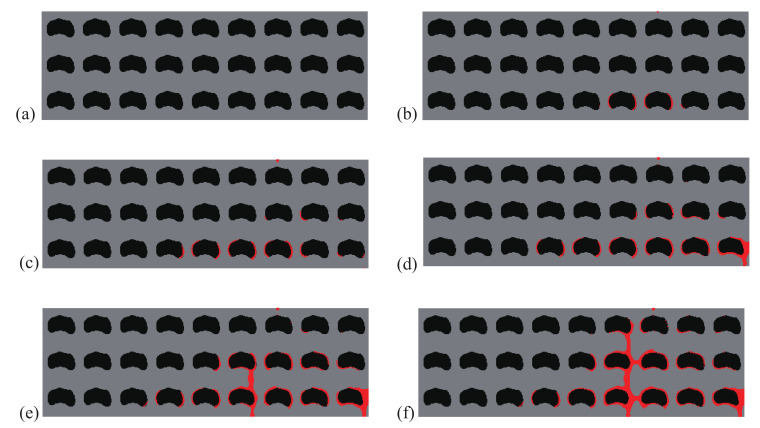
Crack propagation in the periodic composite subjected to non-symmetric three-point bending, optimized design: (**a**) $\bar{U}=0$ mm; (**b**) $\bar{U}=0.17$ mm; (**c**) $\bar{U}=0.24$ mm; (**d**) $\bar{U}=0.33$ mm; (**e**) $\bar{U}=0.39$ mm; (**f**) $\bar{U}=0.44$ mm.

**Figure 13:**
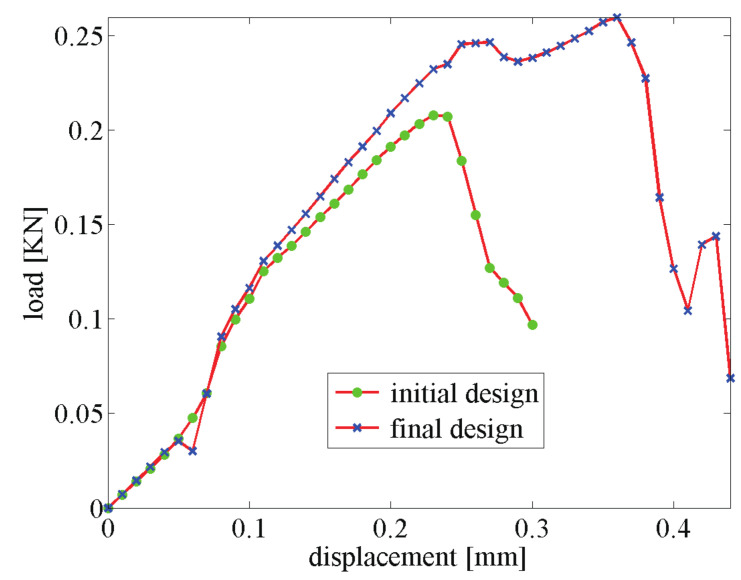
Force–displacement curve of the composites structure subjected to non-symmetric three-point bending.

The crack propagation steps are illustrated for the initial and optimized structures in Figure 11 and Figure 12, respectively. In both cases, cracks initiate from the interface, and then merge in complex crack patterns at the failure step. The crack network is much more complex in the case of the optimized structure and induces a more ductile behavior, as shown in Figure 13. In this example, the gain in fracture resistance for the optimized design is G=100.6%.

### 4.4. Multi-Objective Topology Optimization of a Material Unit Cell with Randomly Distributed Inclusions

**Figure 14:**
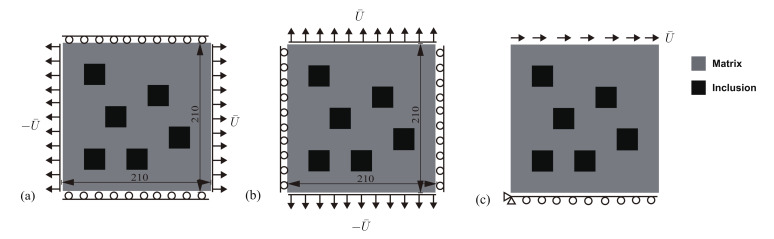
Unit cell of a material containing square inclusions randomly distributed: (**a**) geometry of the initial design and load case 1; (**b**) load case 2; (**c**) load case 3.

In this part, we extend the topology optimization to multi objectives in order to find the optimal topology which maximizes the fracture resistance of a material unit cell possibly subjected to several representative loads. An initial design for the unit cell is described in Figure 14a, consisting of randomly distributed squares, such as their volume fraction is f=12%. Three load cases are considered, whose corresponding boundary conditions are described in Figure 14a–c. Both first loads correspond to traction in the *x*- and *y*-directions, while the third load corresponds to shear.

**Figure 15:**
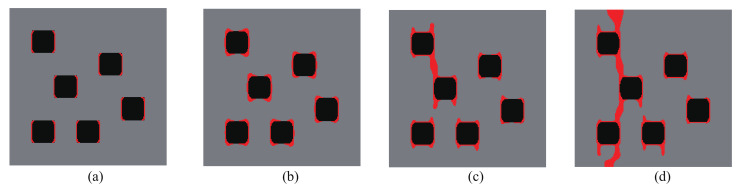
Crack propagation in the initial composite with randomly distributed inclusions subjected to loading case 1: (**a**) $\bar{U}=0.03$ mm; (**b**) $\bar{U}=0.05$ mm; (**c**) $\bar{U}=0.07$ mm; (**d**) $\bar{U}=0.09$ mm.

**Figure 16:**
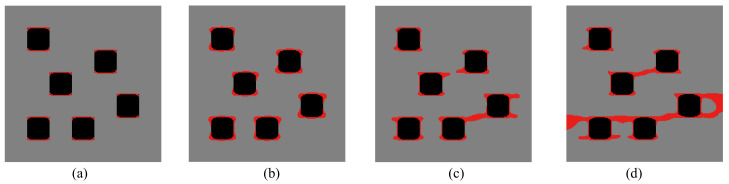
Crack propagation in the initial composite with randomly distributed inclusions subjected to loading case 2: (**a**) $\bar{U}=0.03$ mm; (**b**) $\bar{U}=0.05$ mm; (**c**) $\bar{U}=0.07$ mm; (**d**) $\bar{U}=0.09$ mm.

**Figure 17:**
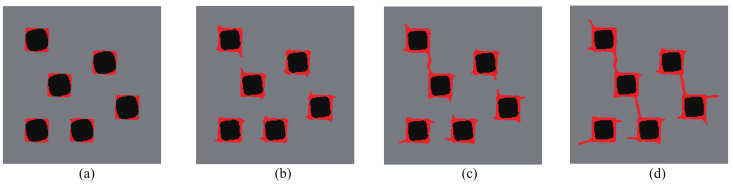
Crack propagation in the initial composite with randomly distributed inclusions subjected to loading case 3: (**a**) $\bar{U}=5$ mm; (**b**) $\bar{U}=10$ mm; (**c**) $\bar{U}=11$ mm; (**d**) $\bar{U}=12$ mm.

Here, the optimization problem is then conducted with respect to the response obtained by these three loads to maximize the objective functions $J=\sum_{i}J_{i}$, where $J_{i}$, *i* = 1:*N* are the objective function defined by (41). The crack paths for the three corresponding loads in the RVE with initial design are shown in Figure 15, Figure 16 and Figure 17, respectively. In all cases, the cracks first initiate from the interfaces and merge into a macro crack which crosses the RVE.

**Figure 18:**
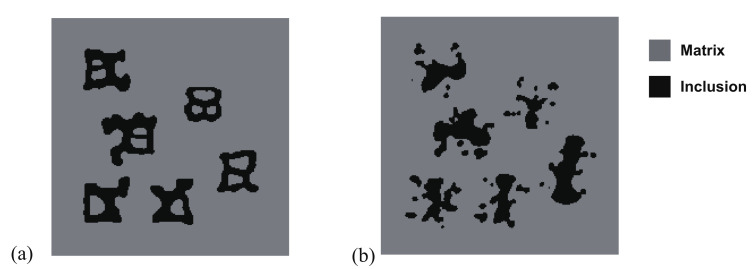
A composite randomly distributed inclusions: (**a**) final design for load cases 1 and 2; (**b**) final design for load cases 1, 2, and 3.

**Figure 19:**
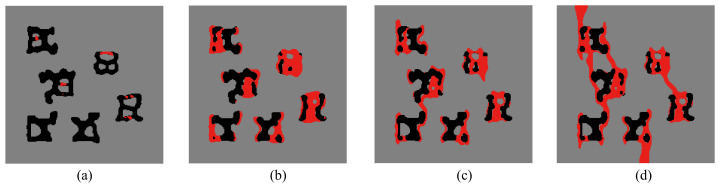
Crack propagation in the final composite without initial cracks subjected to loading case 1: (**a**) $\bar{U}=0.03$ mm; (**b**) $\bar{U}=0.05$ mm; (**c**) $\bar{U}=0.07$ mm; (**d**) $\bar{U}=0.09$ mm.

**Figure 20:**
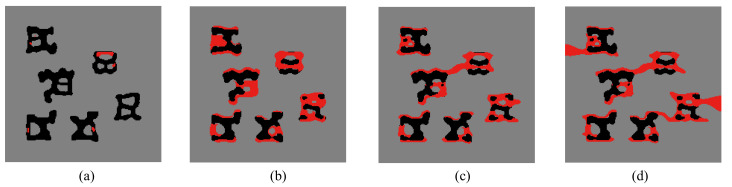
Crack propagation in the final composite without initial cracks subjected to loading case 2: (**a**) $\bar{U}=0.03$ mm; (**b**) $\bar{U}=0.05$ mm; (**c**) $\bar{U}=0.07$ mm; (**d**) $\bar{U}=0.09$ mm.

We first consider only the first two loads in the optimization process, i.e., N=2. The final design is shown in Figure 18a. We can observe a complex, heterogeneous re-distribution of the inclusion phase. The crack paths for the optimized design under loads 1 and 2 are depicted in Figure 19 and Figure 20.

**Figure 21:**
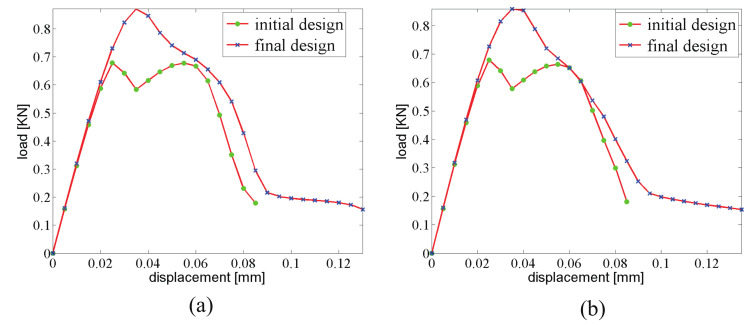
Fracture resistance comparison of two composites with initially randomly distributed inclusions, subjected to (**a**) loading case 1; (**b**) loading case 2.

The force–displacement curves for loads 1 and 2, for initial and optimized designs, are shown in Figure 21a,b, respectively. A gain of G=38.8% improvement of the fracture resistance is achieved as compared with the initial design.

**Figure 22:**
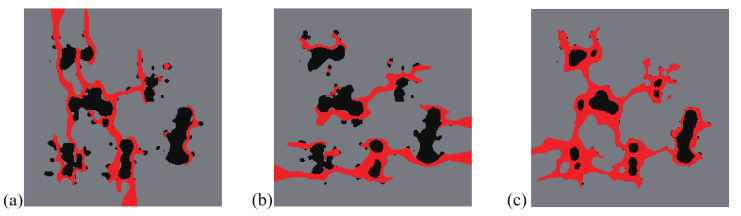
Final crack patterns of: (**a**) final design under load case 1; (**b**) final design under load case 2; (**c**) final design under load case 3.

We now consider the three loads in the optimization process, i.e., N=3. The obtained new distribution of inclusions is shown in Figure 18b. As compared to the previous optimized design, the new topology is more complex here, with a kind of multiscale phase distribution. As shown in Figure 22, the new distribution of inclusions creates a first concentration of cracks within the inclusions, before there is a merging of the different cracks.

**Figure 23:**
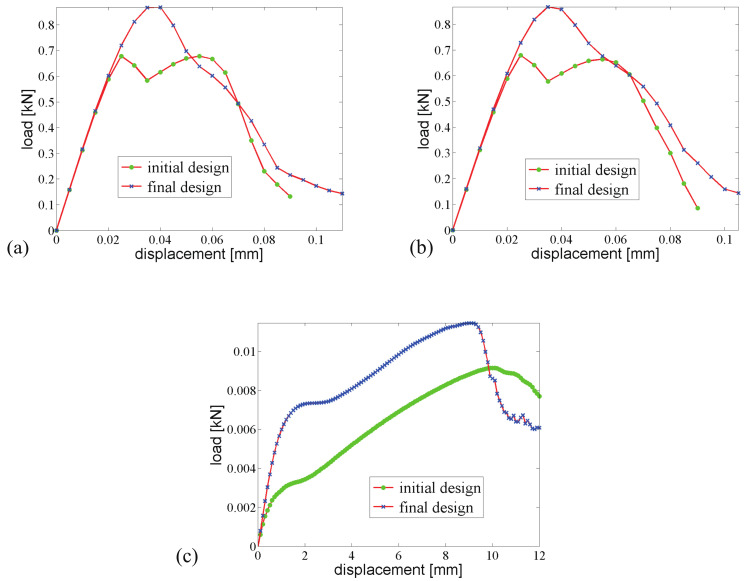
Fracture resistance comparison of two composites with initially randomly distributed inclusions, subjected to (**a**) loading case 1; (**b**) loading case 2; (**c**) loading case 3.

Comparisons between responses of initial and optimized designs for the three loads are depicted in Figure 23. Here, the fracture energy has been improved by 23.8% for case 1, 23.7% for case 2, and 31.2% for case 3. Compared with the design for considering only the first two loading cases in the previous example, the improvement of fracture energy has been reduced; however, we have considered three objectives herein, and the average increase of the required fracture energy is 27.1%.

## 5. Conclusions

In this paper, we have investigated the topology optimization of particle-matrix composites with the objective to maximize its fracture resistance. The framework, initially proposed in [34], combined a phase field method to fracture with interfacial damage and BESO topology optimization, taking into account the full fracture initiation and propagation process, has been extended to periodic composites and multi-objective optimization. We have shown evidence of significant fracture resistance improvements in composite structures under various loading conditions. Size effects have been investigated, showing similar trends as in classical fracture experiments in quasi-brittle structures. More importantly, we have shown that the topology optimization can be performed on a structure with a small number of inclusions at low computational costs. Then, using the optimized topology for larger structures still brings a large gain in the fracture resistance. Finally, we have investigated the effects of multiple loads such that a unit cell of material requires maximizing its fracture for the different possible loads.

## Appendix A. Linearization and Discretization of the Mechanical Problem

Classical FEM discretization is used for discretizing the nodal displacement and nodal displacement increments as:

(A1) $$u\left(x\right)=N\left(x\right)u^{e},\Delta u\left(x\right)=N\left(x\right)\Delta u^{e}$$

where N(x) denotes the matrix of shape functions associated with displacement variables, and $u^{e}$ and $\Delta u^{e}$ are nodal displacement components and nodal incremental displacement components in one element. Furthermore, we have

(A2) $$\left[\varepsilon \left(\Delta u\right)\right]\left(x\right)=B_{u}\left(x\right)\Delta u^{e}$$

where $B_{u}$ is a matrix of shape function derivatives and [ε] is the vector form of the strain tensor ε. The diffuse jump approximation vector and its incremental counterparts can be discretized as

(A3) $$w\left(x\right)=hN\left(x\right){\tilde{B}}_{u}\left(x\right)u^{e},\Delta w\left(x\right)=hN\left(x\right){\tilde{B}}_{u}\left(x\right)\Delta u^{e}$$

where

(A4) $$N=\left[\begin{array}{cccc} n_{1} & n_{2} & 0 & 0\\ 0 & 0 & n_{1} & n_{2} \end{array}\right],$$

with $n_{1}$ and $n_{2}$ being the *x*- and *y*-components of the normal vector to the interface $\Gamma^{I}$, and ${\tilde{B}}_{u}$ is a matrix of shape functions derivatives such that

(A5) $${\left[\begin{array}{c} \frac{\partial u_{1}}{\partial x_{1}}\;\frac{\partial u_{1}}{\partial x_{2}}\;\frac{\partial u_{2}}{\partial x_{1}}\;\frac{\partial u_{2}}{\partial x2} \end{array}\right]}^{T}={\tilde{B}}_{u}u^{e}.$$

The smoothed jump strain at the interfaces is defined by

(A6) $$\left[\bar{\varepsilon }\right]=\left[\begin{array}{c} {\bar{\varepsilon }}_{11}\\ {\bar{\varepsilon }}_{22}\\ \sqrt{2}{\bar{\varepsilon }}_{12} \end{array}\right]=\gamma_{\beta }\left(x\right)\left[\begin{array}{c} w_{1}n_{1}\\ w_{2}n_{2}\\ \frac{1}{\sqrt{2}}\left(w_{1}n_{2}+w_{2}n_{1}\right) \end{array}\right].$$

Then, it yields

(A7) $$\left[\bar{\varepsilon }\left(\Delta u\right)\right]\left(x\right)=h\gamma_{\beta }\left(x\right)M\left(x\right){\tilde{B}}_{u}\left(x\right)\Delta u^{e}$$

with

(A8) $$M=\left[\begin{array}{cccc} n_{1}^{2} & n_{1}n_{2} & 0 & 0\\ 0 & 0 & n_{1}n_{2} & n_{2}^{2}\\ \frac{1}{\sqrt{2}}n_{1}n_{2} & \frac{1}{\sqrt{2}}n_{2}^{2} & \frac{1}{\sqrt{2}}n_{1}^{2} & \frac{1}{\sqrt{2}}n_{1}n_{2} \end{array}\right].$$

Using first-order Taylor expansion of (11) and assuming a staggered solving procedure (i.e., that *d* is fixed when the mechanical problem is solved), the linearized problem is given by

(A9) $$D_{\Delta u}R_{1}\left(u^{k},d\right)=-R_{1}\left(u^{k},d\right)=0,$$

where $D_{v}f\left(.\right)$ is the directional derivative, and $u^{k}$ is the displacement solution from the *k*-th iteration. The displacements at the current iteration are updated according to

(A10) $$u^{k+1}=u^{k}+\Delta u.$$

Using (11) and (A9), we obtain

(A11) $$D_{\Delta u}R\left(u^{k}\right)=\int_{\Omega }\frac{\partial [\sigma^{e}]}{\partial {[\varepsilon }^{e}]}:\left[\varepsilon^{e}\left(\Delta u\right)\right]:\left[\varepsilon^{e}\left(\delta u\right)\right]+\int_{\Omega }\frac{\partial t(w)}{\partial w}:\Delta w:\delta wd\Omega$$

where $\left[\sigma^{e}\right]$, $\left[\varepsilon^{e}\right]$, $\left[\varepsilon^{e}\left(\Delta u\right)\right]$ and $\left[\varepsilon^{e}\left(\delta u\right)\right]$ are the vector forms of their tensorial counterparts. In the above,

(A12) $$\Delta w\left(x\right)=h\nabla \Delta u\left(x\right)\frac{\nabla \phi (x)}{\left||\nabla \phi (x)|\right|}$$

and

(A13) $$\frac{\partial [\sigma^{e}]}{\partial [\varepsilon^{e}]}=C^{tan}\left(u,d\right)=\left[{\left(1-d\right)}^{2}+k\right]\left\{\lambda R^{+}{\left[1\right]}^{T}\left[1\right]+2\mu P^{+}\right\}+\left\{\lambda R^{-}{\left[1\right]}^{T}\left[1\right]+2\mu P^{-}\right\}$$

where $P^{\pm }$ is such that $\left[\varepsilon^{\pm }\right]=P^{\pm }\left[\varepsilon \right]$.

With the above FEM discretization, the linear tangent problem reduces to the following linear system of algebraic equations

(A14) $$K_{tan}\Delta \tilde{u}=-R\left({\tilde{u}}^{k}\right)$$

where

(A15) $$K_{tan}=\int_{\Omega }\left[B_{u}^{T}-h\gamma_{\beta }\left(x\right){\tilde{B}}_{u}^{T}M^{T}\right]C\left(x\right)\left[B_{u}-h\gamma_{\beta }\left(x\right){\tilde{B}}_{u}M\right]d\Omega +\int_{\Omega }h^{2}\gamma_{\beta }\left(x\right){\tilde{B}}_{u}^{T}N^{T}K_{I}N{\tilde{B}}_{u}d\Omega$$

and

(A16) $$\begin{array}{c} R=\int_{\Omega }\left[B_{u}^{T}-h\gamma_{\beta }\left(x\right){\tilde{B}}_{u}^{T}M^{T}\right]C\left(x\right)\left[B_{u}-h\gamma_{\beta }\left(x\right){\tilde{B}}_{u}M\right]{\left(u^{e}\right)}^{k}d\Omega \\ +\int_{\Omega }h\gamma_{\beta }\left(x\right){\tilde{B}}_{u}^{T}N^{T}t\left(w^{k}\right)d\Omega +\int_{\Omega }fN^{T}d\Omega +\int_{\partial \Omega_{F}}\bar{F}N^{T}d\Gamma . \end{array}$$

## Appendix B. Discretization of the Phase Field Problem

Using FEM discretization, the phase field and phase field gradient in one element are approximated by:

(A17) $$d\left(x\right)=N_{d}\left(x\right)d^{e},\nabla d\left(x\right)=B_{d}\left(x\right)d^{e}$$

where $d_{e}$ is a vector of nodal phase field values in one element, and $N_{d}\left(x\right)$ and $B_{d}\left(x\right)$ are matrices of shape functions and of shape functions derivatives associated with the phase field variable, respectively. Introducing the above FEM discretization into the weak form (17), the following linear discrete system of equations can be obtained:

(A18) $$K_{d}\tilde{d}=F_{d}$$

where

(A19) $$K_{d}=\int_{\Omega }\left\{\left(\frac{g_{c}}{\ell }\left(1-\beta \right)+2H\right)N_{d}^{T}N_{d}+\left(1-\beta \right)g_{c}\ell B_{d}^{T}B_{d}\right\}d\Omega$$

and

(A20) $$F_{d}=\int_{\Omega }2N_{d}^{T}H\left(u_{n}\right)d\Omega .$$
